# Enhancing Lower-Body Power in Highly Trained Female Athletes: Effects of Velocity-Based Strength Training

**DOI:** 10.3390/jfmk10040411

**Published:** 2025-10-21

**Authors:** Christoph Schärer, Caterina Barloggio, Jan Seiler

**Affiliations:** Swiss Federal Institute of Sport Magglingen, 2532 Magglingen, Switzerland; caterina29@bluewin.ch (C.B.); jan.seiler@baspo.admin.ch (J.S.)

**Keywords:** explosive strength, female, volleyball, artistic gymnastics, training

## Abstract

**Background:** Only a limited number of studies have examined the effects of short-term, strength–speed-oriented velocity-based training (VBT) on lower-body power in female junior volleyball players and elite female artistic gymnasts. The present study aimed to investigate the impact of a four-week VBT intervention on jump performance and force–velocity characteristics in these athletes. **Methods:** Seven junior female volleyball players (age: 17.4 ± 0.9 years; height: 179.4 ± 6.5 cm; weight: 74.01 ± 3.5 kg) (top-league team members), and seven elite female artistic gymnasts (age: 17.6 ± 2.9 years; height: 159.6 ± 7.2 cm; weight: 59.3 ± 6.3 kg) (National Team members) completed two weekly training sessions for four weeks, each consisting of four sets of six repetitions of parallel back squats (PBSs) and hip thrusts (HTs). Training loads were regulated using barbell velocity targets (PBSs: 0.46–0.72 m/s; HTs: 0.36–0.60 m/s). Pre- and post-intervention assessments included loaded (15–60% body mass) and unloaded squat jumps (SJs) and countermovement jumps (CMJs) to determine peak power output, jump height, and force–velocity profiles. **Results:** Volleyball players showed significant improvements in peak power predominantly during loaded SJs (SJ45%: +5.5%, *p* < 0.01; SJ60%: +5.7%, *p* < 0.05), whereas gymnasts exhibited greater gains in loaded CMJs (CMJ60%: +7.7%, *p* < 0.01). In contrast, unloaded SJ and CMJ performances remained largely unchanged for all athletes. Both groups demonstrated a significantly steeper post-intervention force–velocity profile (*p* < 0.001), indicating an enhanced capacity to produce force at lower movement velocities under external loading. **Conclusions:** Strength–speed-oriented VBT was effective in improving power production under loaded conditions but had limited transfer to unloaded jump performance. These findings highlight the necessity of subsequent training blocks emphasizing high-velocity, sport-specific movements to optimize explosive performance. Future studies should further investigate low-velocity-loss training protocols as a potential means of enhancing unloaded jump outcomes.

## 1. Introduction

In both female individual and team sports, such as artistic gymnastics and volleyball, a high level of mechanical power output during jumping and sprinting is, among other factors, a key determinant of physical performance and competitive success [1]. Female volleyball is characterized predominantly by explosive movements of both the lower and upper limbs, including vertical jumps for blocks, spikes, serves, rapid approach runs, and powerful ball contacts [2,3,4]. In female artistic gymnastics, explosive strength of both the lower and upper limbs is equally essential for performing jump-offs, push-offs, and run-ups across all apparatuses during competitive routines [5,6,7]. Therefore, the enhancement of these physical performance determinants is of practical relevance and plays a key role in female athletic development in both sports. In accordance with the high demands of technical training, it is essential that physical conditioning programs are both time-efficient and effective to minimize the risk of excessive training loads and prevent injuries related to overtraining. However, in female artistic gymnastics and volleyball, as in many other sports, traditional approaches to strength, power, and speed training continue to dominate, while newer, evidence-based conditioning strategies are often underutilized. Furthermore, generic physical preparation programs are frequently implemented instead of individualized strength training protocols.

In contrast to traditional methods for prescribing strength training intensity, such as percentages of the one-repetition maximum (1RM), velocity-based training (VBT) has emerged as a practical and widely implemented approach to optimize strength and power development [8]. VBT uses real-time feedback on movement velocity, allowing precise load monitoring and immediate adjustment during exercise [9,10,11]. This enables individualized load management that accounts for daily fluctuations in strength, speed, and power capacity.

Compared to fixed repetition schemes, which neglect day-to-day variations in neuromuscular performance, VBT offers a more adaptive and effective strategy for resistance training [10]. Real-time feedback not only facilitates more accurate control of training intensity across the continuum from strength to speed but also helps enhance neuromuscular performance by optimizing motor unit recruitment, rate coding, and the rate of force development [12]. Moreover, the motivational aspect of feedback encourages athletes to perform each repetition with maximal intent, thereby improving movement quality and training effectiveness. Furthermore, the linear relationship between external load and the maximal attainable movement velocity—expressed as a percentage of maximal capacity—can be utilized to estimate the actual 1RM in real time during each session and even within individual sets [10,13,14]. Determining the 1RM using traditional testing methods requires the athlete to be in a non-fatigued state and does not allow for daily assessment due to the high physical demands of the test [9,11,15,16]. Continuous monitoring of movement velocity also allows early detection of performance improvements or signs of fatigue, enabling timely adjustments of training loads [14]. Additionally, velocity loss within a set provides valuable information for tailoring training stimuli: higher-rep sets with greater velocity loss (>40%) is associated with muscle hypertrophy and fiber-type transitions, whereas shorter sets with moderate velocity loss (~20%) favors gains in power and maximal strength [11,17]. In the latter case, sets can be terminated once velocity drops below a predefined threshold to maintain execution quality and prevent excessive fatigue.

For these reasons, it appears that the individualized training regulation enabled by VBT is more effective for enhancing strength and jumping performance compared to traditional 1RM methods [18].

To determine whether strength, power, or speed training is most effective for improving mechanical power in the lower limbs, the assessment of an individual force-velocity profile (FVP) can be valuable [1]. This approach involves measuring maximal movement velocity during ballistic actions (e.g., vertical jumps, sprints) under varying loads to characterize the individual linear relationship between force and velocity. To derive specific training recommendations, the FVP leverages the principle that maximal muscular power results from the product of inversely related force and velocity values, and that equivalent submaximal power outputs can be attained through two different combinations of force and velocity [19]. The slope of the linear force–velocity relationship quantifies the extent of force or velocity strengths and weaknesses [8,20,21]. Hence, it indicates whether the athlete would benefit more from strength, power, or speed training in the subsequent training phase. Based on this information, training programs can be individualized to optimize each athlete’s FVP in accordance with the specific demands of their discipline [20,22].

Recently, the FVP has gained increasing popularity among sports professionals for monitoring training progress, as it provides a relatively straightforward basis for deriving precise training recommendations. However, it has been recognized that irrespective of the measurement method used, the estimated parameter of theoretical maximum velocity at zero load exhibits limited reliability [23]. Therefore, it is recommended to directly measure maximal speed, force, and power values across specific sections of the force–velocity continuum and monitor their development over time, rather than relying on extrapolated theoretical parameters. Supporting this practical approach, previous studies applying VBT in volleyball athletes have demonstrated that ballistic resistance training effectively enhances vertical jump performance, particularly in female players [24].

In artistic gymnastics, sport-specific resistance training program are widely used for enhancing muscular power, given that the sport demands high power outputs during short, rapid movements such as sprints and jumps) [5,7]. To date, no studies have been identified that describe a training intervention using VBT with male or female artistic gymnastics athletes. However, findings from other sports suggest that VBT could also be beneficial in artistic gymnastics, particularly for optimizing training load and enhancing explosive strength.

Building on previous research findings, the aim of this study was to examine the effects of a four-week (strength orientated) VBT on lower body power and the FVP performing squat jumps with various external load in junior female volleyball and female elite artistic gymnastics athletes. It was hypothesized that both lower body power under loaded condition and FVP parameters would show improvements following the intervention.

## 2. Materials and Methods

### 2.1. Subjects

The study included seven female volleyball players (age: 17.4 ± 0.9 years; height: 179.4 ± 6.5 cm; weight: 74.01 ± 3.5 kg) members from a top-league team, and seven female artistic gymnastics athletes (age: 17.6 ± 2.9 years; height: 159.6 ± 7.2 cm; weight: 59.3 ± 6.3 kg) from the Swiss National Team. Participant recruitment for both groups and the resulting sample size were primarily determined by availability, representing a convenience sample. With only 14 participants recruited (7 per group), the observed power is approximately 0.45–0.50 for medium effects and ~0.75 for large effects. However, it also provides the opportunity to examine the effectiveness of a training program in athletes with no prior experience in VBT, who are of similar chronological age but differ in training experience. The gymnasts had an average of 12–15 years of training in their sport, whereas the volleyball athletes had an average of 5–7 years. Neither athlete group had prior experience with VBT and had predominantly trained sport-specific lower-body strength exercises throughout their careers. Therefore, this intervention represented a novel training stimulus for both groups. The volleyball players, aged 16–19 years, completed ten training sessions per week (17 h per week), generally three of which were strength training sessions conducted under the supervision of an athletic trainer. Two of these training sessions were exclusively devoted to the VBT protocol implemented in this study, whereas the third strength training session consisted solely of upper-body exercises. The artistic gymnasts, aged 15–23 years, also trained daily under professional conditions with a standardized training load of 25 h per week. Their weekly training routine included twelve sessions: ten sport-specific sessions and two strength training sessions. All athletes were fully informed about the study procedures prior to testing and voluntarily agreed to participate. Informed consent was obtained from all subjects involved in the study. The study was conducted in accordance with the Declaration of Helsinki and approved by the Ethics Committee of the Canton of Bern, Switzerland (KEK) (Project-ID: 2018-00742, 06/072018).

### 2.2. Velocity-Based Training Intervention

**Figure 1:**
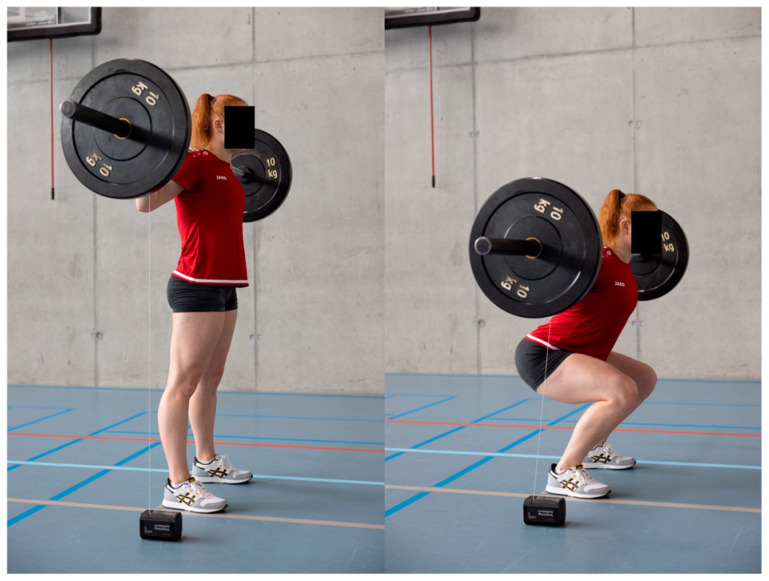
The training exercise parallel back squat was performed using a velocity-based training device.

**Figure 2:**
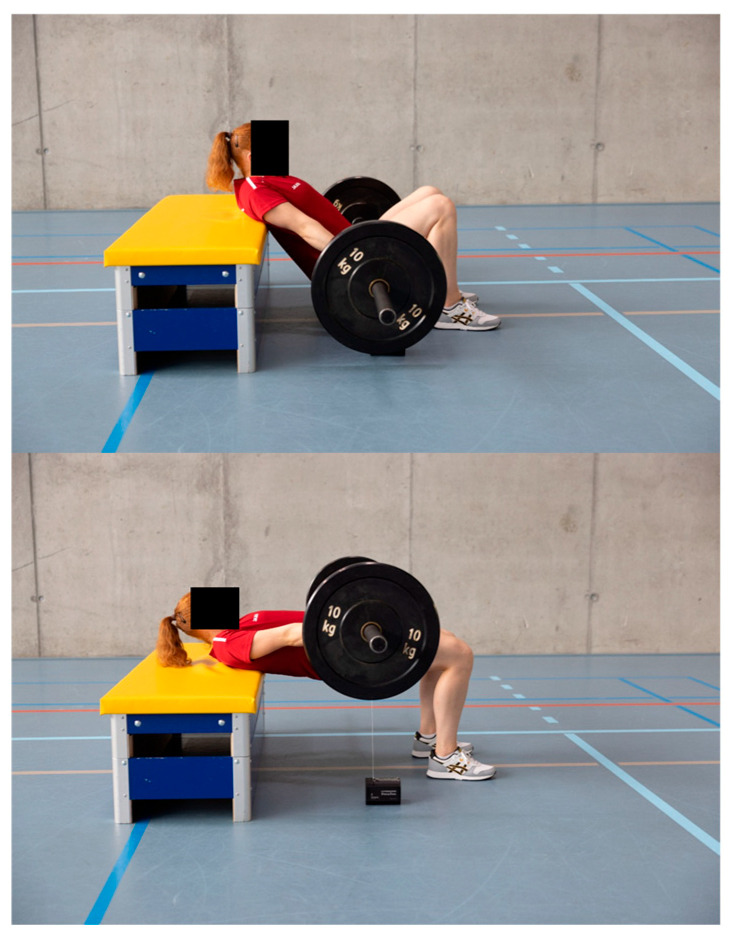
The training exercise hip thrust was performed using a velocity-based training device.

A four-week velocity-based training (VBT) program was implemented for female volleyball and artistic gymnastics athletes. A four-week intervention was implemented, as the seasonal schedule allowed for this duration as the maximum feasible period for the study. During the intervention period, the two VBT sessions replaced the traditional sport-specific strength training sessions for the lower extremities. During each intervention training session, athletes from both sports performed two primary strength exercises—parallel back squats (PBSs, Figure 1) and hip thrusts (HTs, Figure 2), completing four sets of six repetitions per exercise. To ensure training at targeted intensity levels, target mean barbell velocities were established at 0.46–0.72 m/s for PBSs [14] and 0.36–0.60 m/s for HTs [7] (these would have corresponded to approximately 70–90% of 1RM). Velocity thresholds were continuously monitored using a linear position transducer (Gymaware PowerTool, Gymaware, Australia). If the mean velocity of a set exceeded the target, the load was increased by 2.5 kg for the next set; conversely, if the mean velocity fell below the target, the load for the next set was reduced by 2.5 kg. The average velocity per training was calculated to verify adherence to the prescribed velocity zones. A familiarization week was conducted prior to the intervention to ensure correct exercise technique and to prepare participants for the VBT equipment and protocols. An additional week was scheduled for the athletes who had missed sessions, ensuring that all athletes completed 8 training sessions.

### 2.3. Pre- and Posttest

**Figure 3:**
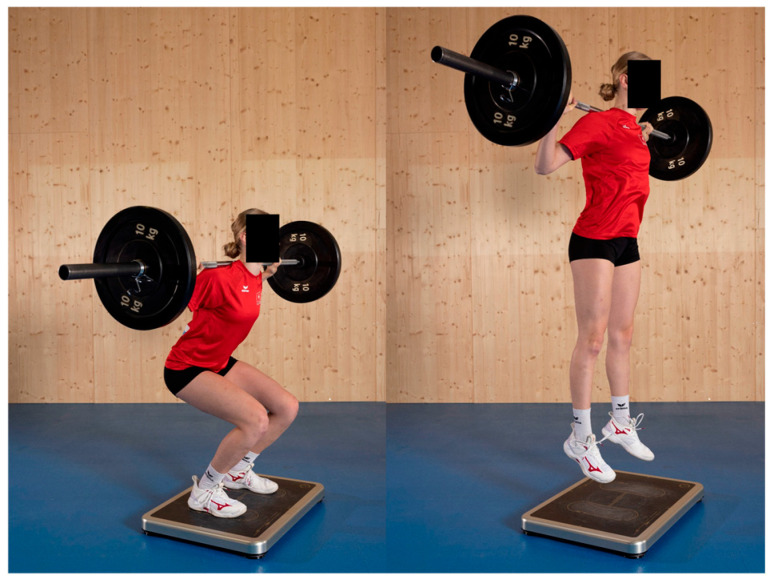
The squat jump test, executed with additional external load, performed on a force plate to assess lower-body power.

One week prior to the first VBT session and one week following the final session, pre- and post-testing were conducted. After a standardized warm-up and the collection of anthropometric data (including body height and weight, leg length in a supine position, and standing leg length at a knee angle of 90°), lower body power was assessed. This included the measurement of maximum relative peak power (Pmax_rel), relative maximal force, and maximal jump height, with mechanical power and jump height being prioritized as widely recognized and easily interpretable metrics for coaches and athletes, ensuring strong practical and sport-specific relevance. These parameters were determined through the performance of multiple countermovement jumps (CMJs) and squat jumps (SJs), both unloaded and with additional external load. The CMJs were performed without load and with a barbell corresponding to 60% of the athlete’s body weight. The SJs were performed without load and with a barbell corresponding to 15%, 30%, 45%, and 60% of body weight. The unloaded jumps were performed first followed by jumps with progressively increasing external load. For each load condition, the two CMJs were performed prior to the SJs. A minimum rest period of 30 s was allowed between jumps. For each load condition, only the jump yielding the highest relative peak power (out of a maximum of three attempts) was used for analysis. A maximum additional load equivalent to 60% of body mass was chosen to ensure that athletes could maintain focus on the take-off phase despite the added weight, while minimizing the risk that apprehension about landing under a greater load would inhibit them from performing a maximal jump. All tests were conducted using a force plate (CYCCESS medical, SPSport, Austria; see Figure 3).

### 2.4. Statistical Analyses

Normal distribution of the data was assessed using the Shapiro–Wilk test. Means and standard deviations were calculated separately for the volleyball and artistic gymnastics groups for the pre- and post-test- and training data. Paired *t*-tests were conducted to compare pre- and post-test results and the loads used in the first and last training sessions. Additionally, effect sizes were calculated using Hedges g. Using the force plate measurements (maximal jump height) from both loaded and unloaded SJs, individual FVPs were determined using the method of Samozino, et al. [25]. To compare the steepness of the mean linear force–velocity (FV) relationships between pre- and posttest, an analysis of covariance (ANCOVA) was performed, effect sizes were calculated using Cohen’s f. Statistical significance was set at *p* < 0.05.

## 3. Results

All athletes completed the pre- and post-assessments, as well as all training sessions, adhering to the prescribed exercises, sets, and repetitions.

### 3.1. Training Monitoring

**Figure 4:**
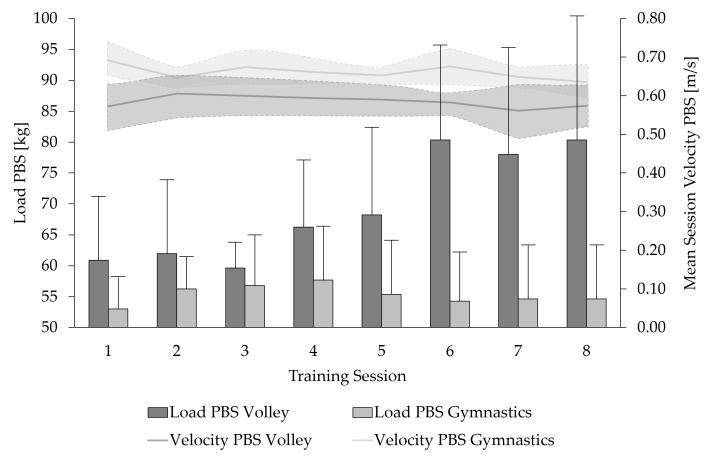
Mean training load and mean session velocity (shaded areas: ± standard deviation) of female junior volleyball players and elite artistic gymnasts during a four-week strength–speed-oriented, velocity-based training intervention in the Parallel Back Squat (PBS) exercise.

**Figure 5:**
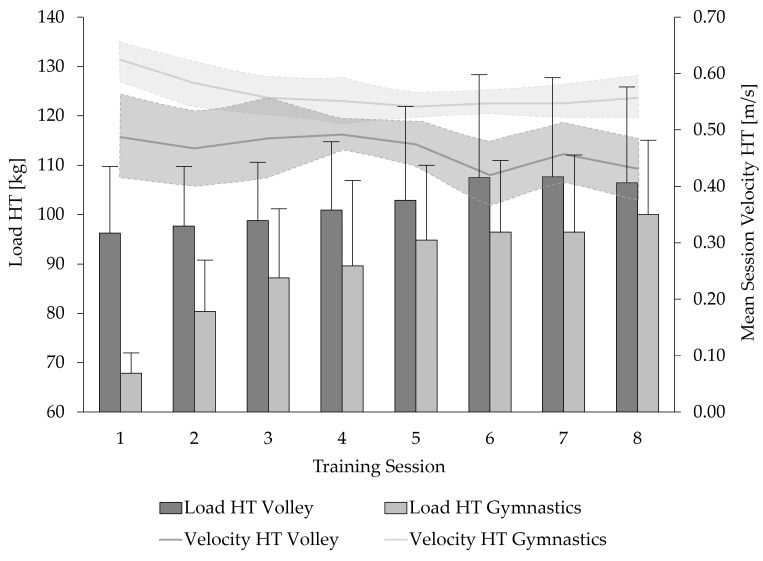
Mean training load and mean session velocity (shaded areas: ± standard deviation) of female junior volleyball players and elite artistic gymnasts during a four-week strength–speed-oriented, velocity-based training intervention in the Hip Thrust (HT) exercise.

Based on mean velocity per training, velocity targets were achieved in 95% of training sessions. Across all training sessions, the gymnasts performed the PBS (+11.5%) and HT (+17.6%) exercises at significantly higher velocities than the volleyball players (*p* < 0.001). Among the volleyball players, training load in the back squat exercise increased significantly by 29% (Session 1: 61.7 ± 10.3 kg; Session 8: 80.4 ± 20.8 kg; *p* < 0.01), whereas no significant change was observed in the hip thrust exercise (Session 1: 96.3 ± 13.9 kg; Session 8: 106.4 ± 20.1 kg; *p* = 0.12). In contrast, gymnasts demonstrated a significant 46.8% increase in training load for the hip thrust exercise (Session 1: 67.9 ± 3.9 kg; Session 8: 100.0 ± 15.3 kg; *p* < 0.001), but no significant change was found for the back squat (Session 1: 53.6 ± 4.3 kg; Session 8: 54.6 ± 8.7 kg; *p* = 0.70; Figure 4 and Figure 5). Following the training intervention, a tendency toward an increase in mean body mass was observed for both groups (volleyball: +1.1 ± 1.2 kg, *p* = 0.05; gymnasts: +0.5 ± 0.75 kg; *p* = 0.06). Detailed individual results are provided in the Supplementary Material (Tables S1–S5).

### 3.2. Lower Body Peak Power

In general, lower-body power increased significantly only under the heaviest loading conditions. Among the volleyball athletes, Pmax_rel increased significantly by 5.5% (*p* = 0.01) and 5.7% (*p* = 0.02) during squat jumps with 45% and 60% additional load, respectively. An increase of 5.7% was also observed during the CMJs with 60% load, although this change was not statistically significant (*p* = 0.11). In artistic gymnasts, a significant increase of 7.7% in Pmax_rel was observed during the CMJs with 60% additional load (*p* = 0.01). Squat jumps with 45% and 60% additional load showed smaller, non-significant improvements of 3.0% (*p* = 0.25) and 3.2% (*p* = 0.22), respectively (Table 1).

Maximum SJ height in volleyball athletes improved significantly with large effect sizes following the four-week training intervention during jumps with 45% and 60% additional load, showing increases of 8.7% (*p* = 0.01) and 10.0% (*p* = 0.02), respectively. Furthermore, an increase of 7.9% (*p* = 0.09) was observed during jumps with 15% additional load; however, this change was not statistically significant (Table 2).

In artistic gymnasts, all squat jumps (SJ) with additional load showed improvements in jump height with large effect sizes (SJ15%: +5.8%, *p* = 0.06; SJ30%: +5.0%, *p* = 0.02; SJ45%: +8.3%, *p* = 0.05; SJ60%: +9.9%, *p* = 0.03). However, only the SJs performed with 30% and 60% additional load demonstrated statistically significant improvements. Notably, the unloaded SJ (0%) and CMJ (0%) conditions showed negligible changes in both groups (*p* < 0.52).

### 3.3. Force-Velocity Profiles

**Figure 6:**
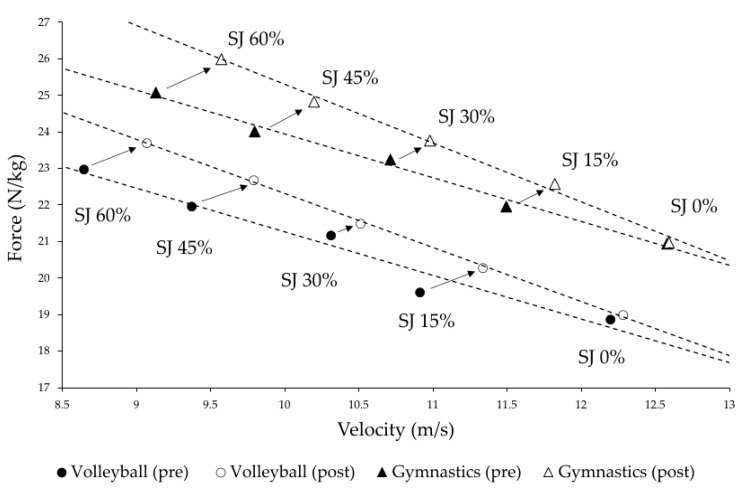
Mean force–velocity profiles of female junior volleyball players and elite artistic gymnasts before (pre) and after (post) a four-week strength-speed-oriented, velocity-based training intervention, determined via squat jumps (SJ) performed without and with additional loads expressed as percentages of body weight.

Force and velocity parameters derived from unloaded and loaded SJ demonstrated a consistent trend of greater improvement with increasing levels of additional load and little to no improvements for unloaded jumps. ANCOVA revealed that the slopes of the mean linear FV relationships differed significantly between pre- and posttest for both groups (volleyball: F = 261.41, *p* < 0.001, f = 4.88; gymnastics: F = 1574.5, *p* < 0.001, f = 11.96). The post hoc power of the ANCOVA was virtually 100% due to the extremely large effect sizes with a sample size of *n* = 7 per group, highlighting the robustness of the findings. Velocity values increased slightly more than force values and similarly across both sports, with velocity improving by up to 4.9% (SJ60%) and force by up to 3.7% (SJ60%), resulting in a significantly steeper linear force–velocity (FV) curve (Figure 6).

## 4. Discussion

This is one of only very few studies that aimed to investigate the effects of a strength-speed-oriented, velocity-based training intervention on maximal lower-body power during unloaded and loaded CMJs and SJs, as well as on force–velocity parameters derived from unloaded and loaded SJs in female athletes. The results demonstrated significant improvements in maximal relative power during loaded squat jumps (45% and 60% of body weight) in volleyball athletes, as well as significantly higher lower-body power during loaded CMJs (60% of body weight) in female artistic gymnasts.

The linear force–velocity relationship derived from unloaded and loaded SJs of volleyball and gymnastics athletes, became significantly steeper after the four-week training intervention. This indicates greater improvements with increased external load. Specifically, movement velocity in SJs increased on average up to +4.9%, which was slightly greater than improvements in force (up to +3.7%). These findings are consistent with previous research demonstrating the effectiveness of VBT for improving load-specific power in female athletes [11,17].

### 4.1. Lower Body Peak Power

The four-week VBT intervention was designed with a strength-speed-oriented focus. Consequently, the target maximal movement velocity for both training exercises—PBS and HT—corresponded to a load intensity of approximately 70–90% of 1RM. This load range is typically associated with the development of maximal strength and power output under loaded conditions [26,27].

As a logical consequence, the intervention primarily enhanced maximal power under loaded condition, which most closely aligns with the movement velocities trained during the strength training program. Therefore, the results observed in our study were consistent with expectations based on the velocity–specificity principle of neuromuscular adaptation after strength training [28].

Generally, the junior volleyball athletes demonstrated slightly greater improvements in lower limb power compared to the elite artistic gymnasts. This may be attributed to the volleyball players’ comparatively lower initial training status. Artistic gymnastics is a discipline characterized by high training volumes beginning in early childhood, whereas volleyball, even at the elite level, typically involves fewer total training hours during developmental stages.

Another potential explanation is the well-established association between maximum strength in the PBS exercise and maximum power output in the SJs and CMJs [27,29]. The greater improvement may therefore lie in the progression observed during the training phase: volleyball players trained at significantly lower velocities, which likely provided a more pronounced strength-training stimulus, and they demonstrated a significant increase in training load for the PBS exercise. In contrast, the gymnasts consistently trained at higher velocities, which may have been more conducive to enhancing muscular power under loaded conditions, as reflected in their improvements in the CMJs. However, gymnasts showed no increase in PBS training load.

Furthermore, it is important to note that the significant improvements observed in the volleyball group were limited to the SJs under the heaviest load condition, while the gymnasts showed significant gains only for the CMJs with the heaviest load. This finding is particularly interesting considering that nearly all jumps performed in artistic gymnastics involve a stretch–shortening cycle [5], similar to CMJs. In contrast, volleyball often includes jump actions more similar to the SJs, particularly during blocking movements. These results underscore that neuromuscular adaptations are highly context-dependent, even when athletes perform the same training program.

The way in which newly acquired neuromuscular potential is expressed appears to be highly specific to the demands of the sport and deeply individual. Such outcomes highlight the importance of contextualizing physical adaptations within the technical and coordinative framework of the practiced sport.

Nonetheless, it must be noted that those jumps most closely resembling sport-specific movement patterns, namely, jumps without additional external load, showed little to no improvement. In contrast to our findings, other authors [11,17] also reported power gains in unloaded jumps following an eight-week VBT intervention in female athletes. A possible explanation for the different findings in our study may be that body mass tended to be higher in both groups following our training intervention. This increase is likely to exert a greater influence on relative muscle power during unloaded jumps than during jumps performed with additional load. Nevertheless, this suggests that despite increases in maximal strength and power under loaded conditions, the transfer to unloaded, high-velocity sport-specific actions remains limited without targeted practice.

From a training perspective, this finding implies that a strength-speed-oriented VBT phase should be followed by a subsequent training block aimed at transferring the newly acquired muscular potential into sport-specific velocity and movement patterns. Such a phase may include plyometric exercises, ballistic movements, or high-velocity jump training closely aligned with the demands of the sport. This could enhance the functional translation of strength gains into muscular power and sport specific performance. It should be noted that the effectiveness of plyometric training in improving power-related parameters in the countermovement jump (CMJ) appears to be strongly dependent on the duration of the intervention. Specifically, interventions lasting longer than 10 weeks have consistently demonstrated significant improvements in CMJ performance, whereas shorter interventions tend to yield smaller adaptations or result in significant effects only in drop jump performance [30,31]. An alternative strategy to enhance muscular strength and power was demonstrated in the study by Achermann, et al. [32]. The investigators employed the exercises squat and deadlift in a two-phase VBT protocol, comprising three weeks of strength-oriented VBT (six sessions, 3 × 5 repetitions, mean concentric velocity: 0.55–0.7 m/s) followed by three weeks of power-oriented VBT (six sessions, 3 × 8 repetitions, mean concentric velocity: 0.8–1.0 m/s). This intervention yielded significant improvements in maximal strength but was also effective at enhancing CMJ and SJ performance without additional external load.

In this context, it should be noted that the velocity loss during the sets was not controlled in our study. Other authors [16,33,34] clearly highlight the benefits of a low velocity loss approach, which has been shown to improve jump performance without additional external load. These studies were mainly conducted with male athletes. Contrary to these studies, Rissanen, Walker, Pareja-Blanco and Häkkinen [11] observed that female athletes tend to achieve greater strength and power gains when using training protocols with a 40% velocity loss threshold compared to programs with a 20% velocity loss threshold. The authors reported that power training programs for women may require a higher training volume than typically used for men to elicit comparable adaptation. To enhance the understanding of female athletes’ adaptations to velocity-based strength training, further research is warranted.

### 4.2. Force–Velocity Profiles

Results from ANCOVA demonstrated a significant increase in the steepness of FV relationships in both groups after the VBT intervention (*p* < 0.001). Force-related parameters increased slightly more than velocity-related parameters during the SJs across the various loading conditions.

In contrast to our findings, other authors [26,35,36] reported significant improvements in both force and velocity following a traditional strength training intervention (five weeks, 4 × 8 repetitions of PBS). The same authors also observed that, after a cluster training intervention (five weeks, 16 × 2 repetitions of PBS), the force–velocity profile shifted more towards a velocity-oriented profile compared with traditional strength training. By comparison, our intervention targeted relatively slow maximal movement velocities. Consequently, the results were largely in line with expectations.

If athletes have limited time to improve their explosive strength, cluster training may offer a practical alternative, as it has the potential to enhance both maximal strength and maximal power within a short period. Nevertheless, in our intervention, athletes were instructed to move (relatively heavily loaded) barbells as fast as possible on each repetition. This intent to maximize movement speed—despite the inherently slow actual velocities—likely provided a high-quality stimulus for both the muscular and nervous systems.

The observed significant shifts in the F–v profile suggest that both athlete groups improved their ability generate force across a spectrum of movement speeds, particularly under substantial mechanical load. However, the near-identical pre- and post-training values for SJ 0% indicate that these adaptations did not transfer to unloaded movements. This highlights a mismatch between strength gains and sport-specific performance potential, reinforcing the concept that adaptations are load- and task-specific, and that transfer to competition-like conditions (e.g., bodyweight jumps) requires subsequent training emphasizing the high-velocity end of the spectrum.

Finally, it is important to note that neither athlete group had prior experience training with a VBT device. The novelty of the stimulus likely contributed to the observed improvements in both strength and movement velocity under high-load conditions consistent with principles of neuromuscular adaptation to novel stimuli [17].

### 4.3. Limitations

No control group could be included in this study, which represents a common limitation in elite sports research, as all athletes aim to benefit from the most effective training interventions. The relatively small sample size (*n* = 7 per group) limits statistical power; however, the participants were of exceptionally high caliber, reflecting the inherent balance between generalizability and the individuality characteristic of elite athletes. Additionally, training loads outside the VBT intervention were not controlled, and variations in sport-specific training volumes may have influenced the results. Data collection was conducted in 2022; however, due to institutional data archiving procedures, it is no longer possible to perform additional analyses. Nevertheless, the findings indicate that, regardless of total training volume, velocity-based training can be highly effective in enhancing sport-specific physical capacities.

## 5. Conclusions

This study demonstrated that a short-term, strength-speed-oriented intervention including the exercises PBS and HT can effectively enhance lower-limb power output in both female junior volleyball players and female elite artistic gymnasts, particularly under loaded conditions. The most pronounced performance improvements were observed with loads that most resembled the training stimulus, with an apparent preference in both groups for the type of jump most prevalent in their respective sports, namely, heavily loaded squat jumps in volleyball athletes and heavily loaded countermovement jumps in gymnasts.

Despite identical training programs, adaptation patterns differed between the two groups, highlighting the influence of sport-specific movement demands and individual neuromuscular characteristics on training outcomes. Force–velocity profiling further revealed a general upward and rightward shift post-intervention, indicating both, improved force and velocity production across a SJ with an additional load. However, the absence of meaningful gains in unloaded jumps points to limited transfer of strength gains to sport-specific loading conditions without targeted, high-velocity training.

These findings underscore the need for a periodized training approach. After a strength-speed-oriented VBT phase, a subsequent block emphasizing velocity- and power-specific modalities—such as plyometrics, ballistic jumps, or high-velocity technical drills—appears essential to fully translate neuromuscular adaptations into functional sport performance. Coaches should therefore consider both the training history and the sport-specific demands of their athletes when designing VBT programs to optimize transfer and maximize performance gains.

## Figures and Tables

**Table 1:** Comparison of lower-body relative maximum peak power (Pmax_rel) during countermovement jumps (CMJ) and squat jumps (SJ), performed without external load (0%) and with additional loads expressed as percentages of body weight (CMJ: 60%; SJ: 15%, 30%, 45%, 60%), in female junior volleyball players and elite artistic gymnasts, measured before (pre) and after (post) a four-week strength-speed-oriented velocity-based training intervention (*: *p* < 0.05; **: *p* < 0.01; g: Hedge’s g).

	Volleyball			Artistic Gymnastics		
(Pmax_rel: W/kg)	Pre	Post	*g*	Pre	Post	*g*
CMJ 0%	46.04 ± 6.63	46.30 ± 5.35	0.10	52.23 ± 3.38	52.65 ± 7.00	0.08
CMJ 60%	40.80 ± 6.25	43.14 ± 5.75	0.67	44.05 ± 5.58	47.46 ± 5.15 **	1.41
SJ 0%	44.64 ± 4.39	43.99 ± 5.68	−0.24	48.65 ± 4.50	49.35 ± 3.75	0.22
SJ 15%	42.46 ± 5.49	43.07 ± 5.64	0.29	45.64 ± 3.30	47.04 ± 3.07	0.63
SJ 30%	42.06 ± 5.13	43.09 ± 5.71	0.41	45.70 ± 3.92	46.17 ± 3.34	0.21
SJ 45%	40.59 ± 4.89	42.81 ± 5.50 **	1.37	43.93 ± 3.75	45.25 ± 3.74	0.45
SJ 60%	39.11 ± 5.10	41.40 ± 4.84 *	1.06	42.99 ± 4.09	44.37 ± 5.30	0.48

**Table 2:** Maximum jump height (s_max) of squat jumps (SJs), performed without external load (0%) and with additional loads expressed as percentages of body weight (CMJ: 60%; SJ: 15%, 30%, 45%, 60%), in female junior volleyball players and elite artistic gymnasts, measured before (pre) and after (post) a four-week strength-speed-oriented velocity-based training intervention (*: *p* < 0.05; g: Hedge’s g).

	Volleyball			Artistic Gymnastics		
(s_max: cm)	Pre	Post	*g*	Pre	Post	*g*
SJ 0%	30.34 ± 4.21	30.76 ± 3.87	0.19	32.24 ± 4.11	32.33 ± 4.91	0.04
SJ 15%	24.29 ± 4.08	26.20 ± 5.91	0.72	26.93 ± 2.75	28.48 ± 3.38	0.81
SJ 30%	21.69 ± 3.33	22.51 ± 3.86	0.45	23.40 ± 2.66	24.58 ± 2.95 *	1.09
SJ 45%	17.90 ± 2.90	19.54 ± 3.18 *	1.21	19.57 ± 2.40	21.20 ± 2.95	0.86
SJ 60%	15.23 ± 2.68	16.76 ± 2.58 *	1.14	17.00 ± 2.66	18.68 ± 2.70 *	0.96

## Data Availability

The raw data supporting the conclusions of this article will be made available by the authors on request.

## Supplementary Materials

The following supporting information can be downloaded at: https://www.mdpi.com/article/10.3390/jfmk10040411/s1. Table S1. Individual results of peak relative power (p_max_rel) of countermovement- and squat jumps without (0%) and with additional load (15–60% of bodyweight (BW)) at pre- and posttest. Table S2. Individual results of maximum jump height (S_max) of squat jumps without (0%) and with additional load (15–60% of bodyweight (BW)) at pre- and posttest. Table S3. Individual results of trainig loads performing parallel back squats (PBS) and hip thrusts (HT) in training sessions 1 to 8. Table S4. Individual results of mean barbell velocitiy performing parallel back squats (PBS) during each session of the training intervention. Table S5. Individual results of mean barbell velocitiy performing hip thrusts (HT) during each session of the training intervention.

## Abbreviations

The following abbreviations are used in this manuscript:

VBT	Velocity Based Training
HT	Hip Thrust
PBS	Parallel Back Squat
CMJ	Countermovement Jump
SJ	Squat Jump
s_max	Maximum Jump Height
Pmax_rel	Relative Peak Power
N	Newton
m	Meter
s	Seconds
FVP	Force–Velocity Profile
