# Supplementary material for: Enhancing Lower-Body Power in Highly Trained Female Athletes: Effects of Velocity-Based Strength Training
Source: J Funct Morphol Kinesiol. 2025 Oct 21;10(4):411. doi: 10.3390/jfmk10040411 (PMC12551051; doi:10.3390/jfmk10040411)
Supplement: Supplementary file 1 [file jfmk-10-00411-s001.zip › jfmk-3860863-supplementary.pdf]

Table S1. Individual results of peak relative power (p_max_rel) of countermovement- and squat jumps without (0%) and with additional load (15% - 60% of bodyweight (BW)) at pre- and posttest.

		CMJ (pmax_rel) [W/kg]				Squat Jump (p_max_rel) [W/kg]									
		Pretest		Posttest		Pretest					Posttest				
**Additional load (% of BW)**		**0%**	**60%**	**0%**	**60%**	**0%**	**15%**	**30%**	**45%**	**60%**	**0%**	**15%**	**30%**	**45%**	**60%**
Athlete 1	Volleyball	38.9	37.5	39.6	38.4	39.7	36.0	36.3	36.2	34.9	39.4	37.6	38.7	37.9	37.5
Athlete 2	Volleyball	43.0	33.1	44.4	41.9	42.0	39.9	40.7	38.6	36.8	43.6	42.1	42.0	42.1	41.2
Athlete 3	Volleyball	57.7	50.9	54.3	53.0	52.0	52.6	51.9	50.2	49.6	51.0	51.5	51.6	52.0	49.6
Athlete 4	Volleyball	40.3	35.6	43.0	39.0	42.3	39.5	39.7	37.4	36.3	39.5	39.1	39.1	38.8	38.2
Athlete 5	Volleyball	45.6	41.4	47.9	40.9	45.6	42.1	40.4	40.4	36.8	45.4	42.9	41.9	42.9	39.8
Athlete 6	Volleyball	45.0	40.4	42.8	39.3	42.1	40.5	39.8	37.7	37.6	37.4	38.2	37.7	37.7	37.0
Athlete 7	Volleyball	51.8	46.7	52.1	49.5	48.8	46.6	45.6	43.6	41.8	51.6	50.1	50.6	48.3	46.5
Athlete 8	Gymnastics	51.7	47.3	48.3	48.3	53.2	49.1	48.6	47.2	45.4	47.5	46.3	44.9	45.8	43.8
Athlete 9	Gymnastics	53.6	49.6	60.3	54.4	52.5	48.4	49.1	47.7	47.3	52.0	49.4	48.6	47.7	50.2
Athlete 10	Gymnastics	48.9	33.4	41.5	37.4	40.4	39.8	38.8	37.4	35.6	42.5	42.1	40.4	38.3	33.7
Athlete 11	Gymnastics	51.2	43.0	54.1	48.2	48.3	45.3	46.7	43.1	43.2	51.1	48.8	49.4	49.1	47.3
Athlete 12	Gymnastics	58.5	47.5	61.8	50.3	51.5	46.7	49.0	45.3	44.9	53.9	49.9	49.2	48.4	47.7
Athlete 13	Gymnastics	48.4	40.6	52.8	46.7	45.6	42.8	42.1	40.9	39.4	48.0	43.8	43.7	43.2	43.5
Athlete 14	Gymnastics	53.2	46.9	50.0	46.9	49.1	47.5	45.6	46.0	45.1	50.5	49.0	47.0	44.2	44.3

Table S2. Individual results of maximum jump height (S_max) of squat jumps without (0%) and with additional load (15% - 60% of bodyweight (BW)) at pre- and posttest.

						Squat Jump (S_max) [cm]									
		Age [y]	Height [cm]	Body Mass [kg]		Pretest					Posttest				
				Pre	Post	0%	15%	30%	45%	60%	0%	15%	30%	45%	60%
Athlete 1	Volleyball	17.1	177.0	77.5	80.4	24.9	19.6	17.3	14.9	12.7	25.9	21.8	18.5	16.6	14.4
Athlete 2	Volleyball	18.9	187.5	69.1	69.8	28.5	22.5	21.2	16.7	14.0	32.1	24.4	22.2	19.4	16.9
Athlete 3	Volleyball	18.3	171.5	73.3	72.9	37.5	31.3	27.5	23.5	20.5	35.0	37.8	27.0	24.2	20.5
Athlete 4	Volleyball	18.6	177.0	77.3	77.5	28.4	22.1	19.8	16.0	13.7	27.9	22.7	20.5	17.1	15.1
Athlete 5	Volleyball	16.4	185.0	71.2	73.4	28.8	22.1	20.8	17.9	13.9	29.3	23.8	21.2	19.1	15.4
Athlete 6	Volleyball	16.7	185.0	77.7	78.2	30.0	24.1	20.7	16.6	14.8	28.7	22.3	19.5	16.8	14.8
Athlete 7	Volleyball	16.0	172.5	72.1	73.7	34.3	28.3	24.5	19.7	17.0	36.4	30.6	28.7	23.6	20.2
Athlete 8	Gymnastics	17.9	154.0	61.4	62.9	32.1	28.7	24.4	20.5	20.3	28.9	28.1	24.8	21.3	19.0
Athlete 9	Gymnastics	15.5	150.0	50.7	50.5	35.9	28.1	24.7	21.0	18.6	38.1	31.2	27.5	24.8	21.9
Athlete 10	Gymnastics	14.8	158.0	60.8	61.1	25.8	22.2	19.2	15.0	12.0	24.6	22.2	19.9	15.4	13.7
Athlete 11	Gymnastics	17.1	158.0	56.9	56.7	31.7	25.5	22.0	18.1	16.3	33.6	30.1	24.5	21.6	18.8
Athlete 12	Gymnastics	17.6	172.0	70.3	71.6	38.7	30.9	27.7	21.3	18.4	38.3	32.6	28.7	23.5	21.4
Athlete 13	Gymnastics	16.6	165.0	60.6	61.6	31.0	26.2	22.1	19.2	15.9	30.8	27.2	22.4	21.1	18.0
Athlete 14	Gymnastics	23.7	160.0	54.2	54.0	30.5	26.9	23.7	21.9	17.5	32.0	28.0	24.3	20.7	17.9

Table S3. Individual results of trainig loads performing parallel back squats (PBS) and hip thrusts (HT) in training sessions 1 to 8.

		Load PBS [kg]								Load HT [kg]							
	**Session**	1	2	3	4	5	6	7	8	1	2	3	4	5	6	7	8
Athlete 1	Volleyball	57.5	52.5	60	67.5	70	65	70	72.5	83.8	91.25	93.75	96.25	96.25	105	111.25	110.0
Athlete 2	Volleyball	55.0	55	55	55	55	63.75	65	62.5	80.0	80	80	80	80	86.25	85	78.8
Athlete 3	Volleyball	83.8	86.25	60	85	95	110	112.5	122.5	120.0	118.75	120	125	141.25	150	146.25	140.0
Athlete 4	Volleyball	62.5	65	60	67.5	62.5	80	65	67.5	93.8	97.5	100	100	100	100	97.5	100.0
Athlete 5	Volleyball	55.0	55	55	55	55	80	70	70.0	90.0	90	93.75	92.5	90	90	90	90.0
Athlete 6	Volleyball	55.0	55	60	60	63.75	80	75	75.0	102.5	102.5	100	103.75	105	106.25	110	110.0
Athlete 7	Volleyball	63.3	65	67.5	73.75	76.25	83.75	88.75	92.5	103.8	103.75	103.75	108.75	107.5	115	113.75	116.3
Athlete 8	Gymnastics	56.7	61.25	62.5	62.5	60	50	50	50.0	71.8	85	97.5	101.25	101.25	101.25	105	110.0
Athlete 9	Gymnastics	53.8	55	56.25	55	53.75	50	50	50.0	71.0	75	88.75	82.5	92.5	92.5	80	95.0
Athlete 10	Gymnastics	53.3	57.5	60	60	60	60	60	60.0	66.0	87.5	90	90	90	92.5	95	95.0
Athlete 11	Gymnastics	55.0	61.25	60	62.5	60	50	50	50.0	68.0	85	97.5	101.25	110	98.75	105	110.0
Athlete 12	Gymnastics	60.5	60	60	68.75	66.25	70	72.5	72.5	71.8	95	101.25	115	115	120	115	115.0
Athlete 13	Gymnastics	48.8	48.75	48.75	45	45	50	50	50.0	65.0	65	65	67.5	85	100	105	105.0
Athlete 14	Gymnastics	47.5	50	50	50	42.5	50	50	50.0	61.7	70	70	70	70	70	70	70.0

Table S4. Individual results of mean barbell velocitiy performing parallel back squats (PBS) during each session of the training intervention.

		Mean velocity PBS per training [m/s]							
	**Session**	**1**	**2**	**3**	**4**	**5**	**6**	**7**	**8**
Athlete 1	Volleyball	0.63	0.71	0.68	0.66	0.57	0.60	0.67	0.61
Athlete 2	Volleyball	0.50	0.52	0.52	0.53	0.58	0.61	0.55	0.51
Athlete 3	Volleyball	0.64	0.63	0.62	0.54	0.65	0.58	0.57	0.60
Athlete 4	Volleyball	0.53	0.60	0.61	0.59	0.54	0.60	0.60	0.52
Athlete 5	Volleyball	0.52	0.58	0.61	0.62	0.65	0.59	0.44	0.65
Athlete 6	Volleyball	0.56	0.58	0.61	0.59	0.56	0.52	0.51	0.52
Athlete 7	Volleyball	0.63	0.62	0.55	0.63	0.58	0.58	0.59	0.61
Athlete 8	Gymnastics	0.75	0.65	0.74	0.66	0.66	0.7	0.65	0.68
Athlete 9	Gymnastics	0.62	0.65	0.61	0.62	0.64	0.7	0.64	0.63
Athlete 10	Gymnastics	0.71	0.61	0.64	0.63	0.62	0.59	0.6	0.57
Athlete 11	Gymnastics	0.7	0.65	0.69	0.67	0.66	0.7	0.65	0.68
Athlete 12	Gymnastics	0.7	0.64	0.67	0.7	0.68	0.67	0.66	0.65
Athlete 13	Gymnastics	0.68	0.65	0.69	0.71	0.67	0.7	0.65	0.6
Athlete 14	Gymnastics	0.68	0.68	0.68	0.64	0.64	0.67	0.69	0.64

Table S5. Individual results of mean barbell velocitiy performing hip thrusts (HT) during each session of the training intervention.

		Mean velocity HT per training [m/s]							
	**Session**	**1**	**2**	**3**	**4**	**5**	**6**	**7**	**8**
Athlete 1	Volleyball	0.63	0.58	0.34	0.47	0.56	0.53	0.55	0.48
Athlete 2	Volleyball	0.44	0.40	0.54	0.46	0.43	0.39	0.50	0.43
Athlete 3	Volleyball	0.55	0.43	0.50	0.51	0.45	0.32	0.44	0.34
Athlete 4	Volleyball	0.45	0.39	0.41	0.48	0.45	0.42	0.38	0.48
Athlete 5	Volleyball	0.42	0.50	0.53	0.52	0.47	0.45	0.47	0.44
Athlete 6	Volleyball	0.50	0.47	0.52	0.53	0.49	0.46	0.44	0.37
Athlete 7	Volleyball	0.42	0.50	0.48	0.47	0.47	0.48	0.42	0.48
Athlete 8	Gymnastics	0.58	0.55	0.5	0.52	0.53	0.54	0.52	0.54
Athlete 9	Gymnastics	0.65	0.61	0.59	0.54	0.52	0.58	0.59	0.6
Athlete 10	Gymnastics	0.59	0.6	0.55	0.6	0.59	0.51	0.54	0.52
Athlete 11	Gymnastics	0.69	0.65	0.6	0.52	0.53	0.57	0.52	0.54
Athlete 12	Gymnastics	0.6	0.5	0.59	0.49	0.55	0.53	0.54	0.5
Athlete 13	Gymnastics	0.59	0.57	0.51	0.6	0.53	0.54	0.52	0.59
Athlete 14	Gymnastics	0.67	0.6	0.56	0.59	0.54	0.56	0.6	0.61
